# Integration and Assessment of ChatGPT in Medical Case Reporting: A Multifaceted Approach

**DOI:** 10.3390/ejihpe14040057

**Published:** 2024-03-30

**Authors:** Kuan-Chen Lin, Tsung-An Chen, Ming-Hwai Lin, Yu-Chun Chen, Tzeng-Ji Chen

**Affiliations:** 1 Department of Family Medicine, Taipei Veterans General Hospital, No. 201, Sec. 2, Shih-Pai Road, Taipei 11217, Taiwan; s10001002@gm.ym.edu.tw (K.-C.L.); firen8056@gmail.com (T.-A.C.); minghwai@gmail.com (M.-H.L.); 2 School of Medicine, National Yang Ming Chiao Tung University, Taipei 30010, Taiwan; 3 Institute of Hospital and Health Care Administration, School of Medicine, National Yang Ming Chiao Tung University, Taipei 30010, Taiwan; 4 Big Data Center, Taipei Veterans General Hospital, Taipei 11217, Taiwan; 5 Department of Family Medicine, Taipei Veterans General Hospital Hsinchu Branch, No. 81, Sec. 1, Zhongfeng Road, Zhudong Township, Hsinchu 310403, Taiwan; 6 Department of Post-Baccalaureate Medicine, National Chung Hsing University, No. 145, Xingda Road, South District, Taichung 402202, Taiwan

**Keywords:** artificial intelligence, case reports, LLM, clinical thinking, medical writing, health science

## Abstract

ChatGPT, a large language model, has gained significance in medical writing, particularly in case reports that document the course of an illness. This article explores the integration of ChatGPT and how ChatGPT shapes the process, product, and politics of medical writing in the real world. We conducted a bibliometric analysis on case reports utilizing ChatGPT and indexed in PubMed, encompassing publication information. Furthermore, an in-depth analysis was conducted to categorize the applications and limitations of ChatGPT and the publication trend of application categories. A total of 66 case reports utilizing ChatGPT were identified, with a predominant preference for the online version and English input by the authors. The prevalent application categories were information retrieval and content generation. Notably, this trend remained consistent across different months. Within the subset of 32 articles addressing ChatGPT limitations in case report writing, concerns related to inaccuracies and a lack of clinical context were prominently emphasized. This pointed out the important role of clinical thinking and professional expertise, representing the foundational tenets of medical education, while also accentuating the distinction between physicians and generative artificial intelligence.

## 1. Key Messages

1. ChatGPT was mainly used for content generation and medical information retrieval in 64 case reports.
2. Over 50% of ChatGPT applications began with information retrieval, later evolving for various needs.
3. About 70% of authors noted inaccuracies. The second most commonly mentioned limitation was clinical context issues.
4. ChatGPT was applied across various medical domains, indicating its widespread applicability.
5. Effective but requires oversight; enhancements needed for accuracy and context.

## 2. Introduction

In November 2022, the advent of ChatGPT, a paradigm-shifting natural language processing model, heralded a seminal transformation across diverse domains, with profound implications for the healthcare sector. Traditional artificial intelligence, also known as symbolic logic, operates through sequential logical inference, requiring the identification of specific features before training can commence. In practice, it often relies on professional assistance to construct logical rules and establish databases, operating as expert systems in various fields. However, its reliance on regular maintenance and environmental constraints imposes limitations on its scalability. In contrast, the perceptron paradigm, which evolved into neural networks, draws its main concepts from the neuronal transmission patterns of biological organisms and can accommodate multiple input factors. The practical development process requires a significant amount of learning data and is suitable for complex decision-making patterns, such as deep learning. Built on a multilayered neural network framework, deep learning enables automated feature recognition through extensive database training, achieving highly complex outcomes. ChatGPT, short for Generative Pre-trained Transformer, is a deep learning-based generative language model. During its training process, it employs multi-layer attention mechanisms to process input content and learns the structure and semantics of language through pre-training. The main feature of ChatGPT is its ability to understand and produce natural language text due to its extensive training across various text domains and numerous parameters. Therefore, the proficiency of ChatGPT in comprehending and generating text has led to a wide range of applications, including language translation, information summarization, and text classification [1,2,3]. This introduction has, in essence, sparked a revolution in the document creation industry.

Beyond its application by the general populace and in business enterprises [4], the healthcare sector has exhibited a great interest in exploring its potential [5,6,7,8,9,10,11,12,13] and has explored the applicability of ChatGPT in the context of medical record management, including medical records [14,15], surgical reports [16,17], radiology findings [18,19,20], and discharge summaries [17,21] in the realm of clinical practice. Traditional medical record composition requires the meticulous cross-referencing of textual content scattered across disparate files and the systematic organization of inpatient progress along chronological timelines. Likewise, the creation of surgical reports, radiology findings, and related documents need the rapid generation of templates and the elimination of superfluous verbiage. Furthermore, significant emphasis has been placed on the post hoc generation of case reports based on clinical data.

However, alongside the potential benefits, concerns have arisen regarding the accuracy of content generated by ChatGPT [22,23,24], issues of academic ethics [25,26,27,28,29,30], privacy, security [31,32], and potential biases in training databases [33]. These concerns are crucial, considering the sensitive nature of medical data and the need for precision in medical documentation.

Case reports play a vital role in medical literature, offering insights into unique clinical experiences, treatment responses, and emerging diseases. These comprehensive, real-life cases not only offer new directions for research and treatment, but also aid in clinical reasoning when confronted with similar symptoms, thereby broadening the possibilities for differential diagnosis and management. They necessitate a detailed, chronological narrative description of a patient’s medical journey, demanding meticulous organization and textual articulation. Given the substantial time and effort required to compose detailed medical records and reports, the clinical medicine sphere is earnestly exploring the potential of ChatGPT in the realm of case report composition. In this light, ChatGPT’s proficiency in text organization and content generation appears highly promising. However, the current landscape lacks comprehensive research on the specific methodologies by which clinicians utilize ChatGPT for writing case reports and the depth of its impact on the quality and accuracy of these reports.

Therefore, the purpose of this study is to understand, approximately six months post-release of ChatGPT, how the medical community has adopted this tool in medical case reporting. We aim to provide a comprehensive analysis of ChatGPT’s role in medical case reporting, offering insights into its utility, limitations, and implications for accuracy and ethics in medical practice. This exploration is vital to ensure that while leveraging the capabilities of ChatGPT, medical professionals maintain the precision and accuracy essential in medical writing.

## 3. Materials and Methods

### 3.1. Search Strategy for Case Report Using ChatGPT

**Figure 1:**
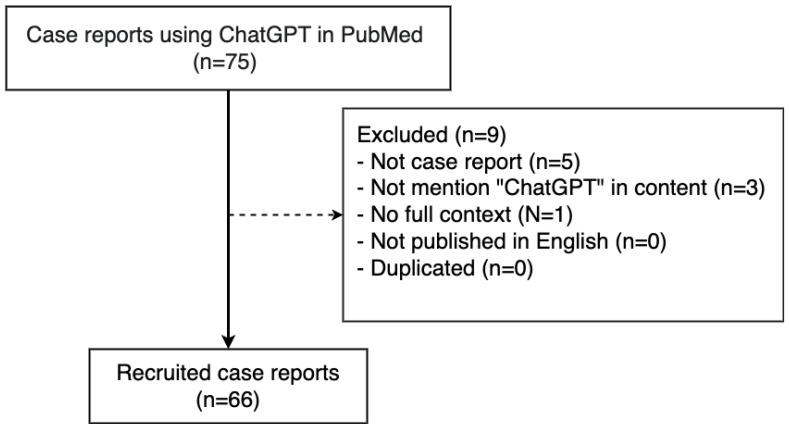
Flowchart of selection of case report using ChatGPT in PubMed.

This bibliometric analysis of case reports facilitated by ChatGPT was rigorously conducted using the PubMed database. The study encompassed the timeframe from 1 December 2022 to 31 December 2023. To identify case reports, our search used the keywords “case report” OR “case reports.” The specific search terms executed within the PubMed database were as follows: (“ChatGPT” OR “Chat GPT” OR “Chat GPT 3.5” OR “GPT 3.5” OR “GPT-3.5” OR “ChatGPT 3.5” OR “ChatGPT-3.5” OR “GPT-4” OR “GPT4” OR “GPT 4” OR “ChatGPT-4” OR “ChatGPT4” OR “ChatGPT 4”) AND (“Case Reports”[pt] OR (case report) OR (case reports)). The exclusion criteria encompassed research articles that did not meet the definition of a case report, publications that were not disseminated in English, duplicate entries, lack of comprehensive contextual information, or absence of the term “ChatGPT” in their textual content. After a thorough review of each article, a total of 66 articles were finally included in our analysis (Figure 1).

### 3.2. Data Extraction

Case reports involving ChatGPT, as archived in PubMed, were identified, and data including publication title, author names, country of affiliation of the first author, publication date, journal name, field of publication, full text, and keywords were extracted. After a thorough review of each article, we performed further categorization based on the language used for ChatGPT input, whether the application of ChatGPT was mentioned, whether any limitations were reported during the research, the specific version of ChatGPT or any extensions employed, the field of the articles, and the publication month.

### 3.3. Data Analysis

To delve deeper into the application and limitations of ChatGPT in the context of case reports, we categorized these aspects into distinct groups. During the initial phase of exploring ChatGPT’s capabilities, the public lacked a robust corpus of the literature describing explicit categorizations. After a careful review of the content of the included articles, our team members reached a consensus on the classifications of applications and limitations. Two independent researchers then conducted a rigorous analysis of the full texts of the included articles, systematically identifying and assigning the use of the application categories or the mention of limitation elements for each study. The findings were presented in terms of the number of articles and their proportional distribution within each specialty. To ensure accuracy and consistency, any discrepancies in the assessments were adjudicated by an impartial third party, thus guaranteeing the reliability and validity of our results.

In our attempt to comprehensively investigate the utilization of ChatGPT, we categorized its functionalities into several classes, including context generation, information retrieval, editing, structural presentation, result interpretation, and addressing open-ended question.

The “content generation” category can be further subdivided into functions such as generating the title, draft/content, outline, introduction, result, conclusion, discussion, and other sections, which will be listed separately. For instance, if the author inputs into ChatGPT such as “write an abstract”, “what title would you suggest for this”, or “generate 5 MeSH keywords for this article, including ChatGPT”, they would fall under the category of content generation. Subsequently, an in-depth analysis would be conducted based on the query.

If the author’s input commands are aimed at obtaining information from ChatGPT, similar to querying a search engine, they would be categorized under “information retrieval”. This category encompasses requests such as providing a list of citations, adding relevant information from references, and offering a specific summary of editorials and commentaries. Examples of original input commands include: “What is the possible diagnosis?”, “What are other medical conditions that might cause these patient’s symptoms?”, “How should we assess this patient?”, “Which medications should be used to treat this patient?”, “Generate a paragraph about a common complication of watchman fix implantation”, “What is the importance of reporting variants of uncertain significance in medical literature”, “Complication of untreated Amyand’s hernia, write a brief summary with reference”, and “Provide a brief overview of what hyperbaric oxygen therapy is and its common indications”.

The category of “editing” comprises paraphrasing or rewriting in a case presentation style, such as converting the paragraph to bullet points. For instance, an original input command could be: “Is there a better term than psychiatric illness that may have less stigma associated with it?”

When authors request ChatGPT to provide an outline, structural guidance, or suggestions, it is categorized as “structural presentation”. Examples of original input commands include “Write a manuscript outline on a certain topic”, “Help me write a case report” by providing relevant information, or “I want to participate in the Turing test with you, could you do it for me”, etc.

The category of “result interpretation” includes tasks such as merging data, creating tables, sorting and managing references and citations, formatting references according to instructions, identifying recurrent PMIDs and labeling them, interpreting the significance of the given lab data, reorganizing multiple MRI results chronologically and presenting paragraphs in case report style based on examination results, imaging reports, or patient medical records.

The “open-ended question category” consists of responses to queries such as “Generate future research design or novel treatments for choriocarcinoma syndrome…” and, in situations where drug quantities are not involved, provides customized recommendations that address identified concerns and abnormalities.

As for the limitations of ChatGPT in case reports, we have categorized these limitations into the following categories: inaccuracy (also known as artificial intelligence hallucination), lack of clinical context, outdated information, lack of creativity, bias, privacy concerns, ethical issues, unreliable sources, and technical issues. The technical issue category encompasses instances of system crashes and failure to execute commands as instructed.

## 4. Results

### 4.1. Characteristics of Case Reports Using ChatGPT

A total of 66 case reports using ChatGPT were included in this study from November 2022 to December 2023. Among the 66 articles, 65 (98.5%) used ChatGPT with English input, except one using Spanish. Out of these, all articles mentioned how ChatGPT was employed. Additionally, 32 articles (48.5%) discussed the limitations they encountered when using ChatGPT. Out of the 66 papers, 64 (97%) only used the web-based version of ChatGPT, one article mentioned using an extended function, one conducted a comparison between the web version ChatGPT with the desktop version, GPT zero, and the remaining two articles made no mention of this aspect (Table 1).

**Figure 2:**
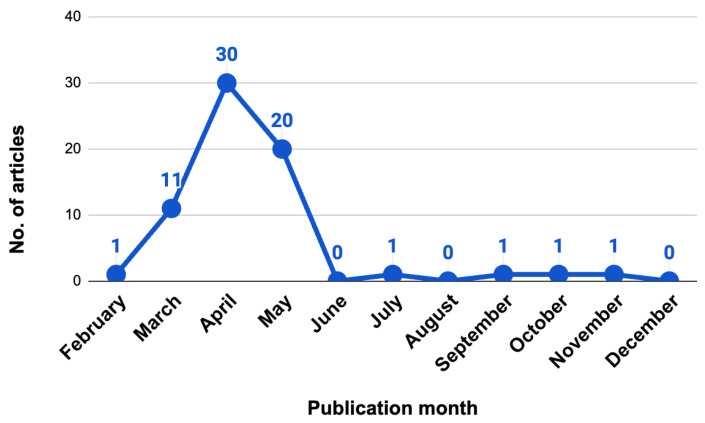
Publication trends of case reports utilizing ChatGPT till December 2023.

Among the 66 articles, there was a diversified distribution among medical specialties. Overall, 21.2% were published in internal medicine, 9.1% in otolaryngology, 7.6% in dermatology, while neurosurgery and cardiology each accounted for 6.1%. The remaining publications accounted for 50% of the total. Of these 66 articles, an overwhelming majority of 63 (95.5%) were published in *Cureus*, while the remaining three articles were disseminated in different scientific outlets, namely *Archivos de la Sociedad Española de Oftalmología*, *Clinical Case Reports,* and *eNeurologicalSci*. The temporal distribution of publications spanned from February to December, with the following breakdown: a single article (1.5%) was published in February, followed by 11 articles (16.7%) in March, a substantial contribution of 30 articles (45.5%) in April, and 20 articles (30.3%) in May. The following months, July, September, October and November, each saw the publication of a single article (1.5% per month). The publication months of case reports using ChatGPT was depicted in Figure 2.

### 4.2. Application of ChatGPT Mentioned in Case Reports

Among the 66 articles that mentioned how ChatGPT was applied, more than half of these articles (N = 36, 55.7%) relied on ChatGPT’s medical information retrieval functionality. This encompassed a variety of tasks, including seeking explanations of medical terms, requesting specific topic-related information, instructing ChatGPT to locate reference sources, obtaining statistical data, seeking differential diagnoses, and receiving treatment recommendations.

**Figure 3:**
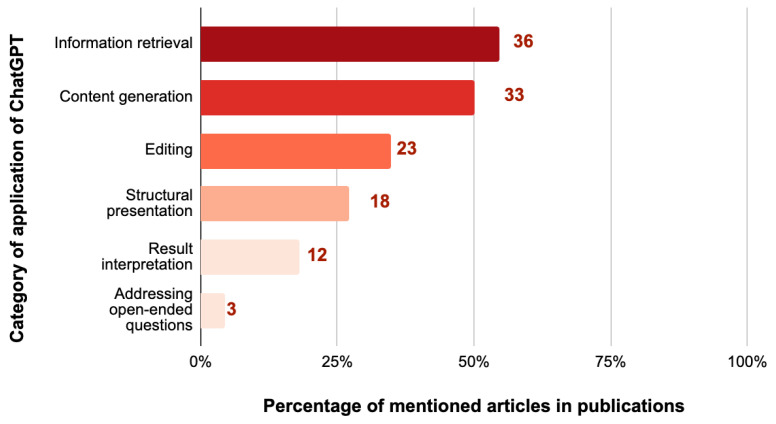
Percentage and number of case report articles categorized by the application of ChatGPT in case report writing (N = 66).

Furthermore, half of the articles (N = 33, 50%) utilized ChatGPT for content generation, ranging from creating titles to drafting sections. About one-third provided ChatGPT with pre-written content, asking it to refine grammar and rewrite text (N = 23, 34.8%). A significant proportion, over a quarter (N = 18, 27.3%), used ChatGPT for structural presentation tasks. This involved generating case report outlines and transforming content into structured formats. Around 18% of the articles used ChatGPT for result interpretation (N = 12), a process that entailed converting original content into tables or arranging reference materials in specific formats or sequences. In contrast, only three articles (4.5%) requested ChatGPT to address open-ended questions, such as soliciting feedback or exploring potential future treatment modalities (Figure 3).

Of the 33 articles that used ChatGPT for content generation, approximately 39% used it exclusively for generating content or drafts, while 36% used it specifically for crafting introductions. About one-third of these articles used ChatGPT for generating titles and abstracts. A smaller proportion, 6.1%, applied it to generate results, whereas 27% employed it for composing conclusions, and 21% used it to shape discussions.

**Figure 4:**
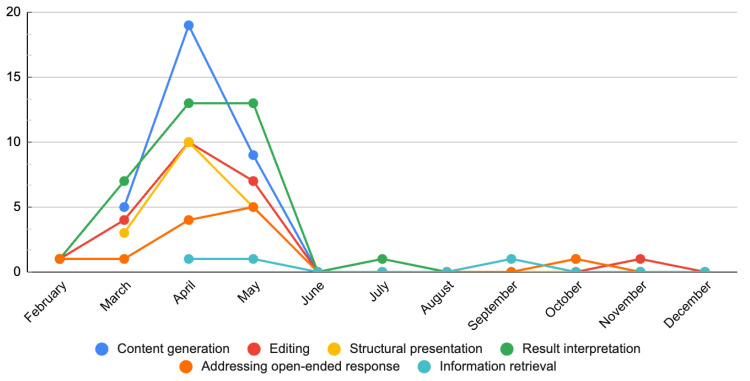
Publication numbers by application category from February to December 2023.

The number of articles published for each month based on each ChatGPT application category was presented in Figure 4. Articles submitted in February made use of information retrieval, editing, and result interpretation. Articles utilizing content generation and structural presentation were found since March. Articles addressing open-ended response emerged since April. Interestingly, the number of articles using content generation, editing, and structural presentation functions peaked in April and slightly declined in May. Conversely, applications involving information retrieval, interpretation of results, and handling of open-ended questions showed a moderate increase or remained relatively stable until the month of May. Despite the limited number of articles published in the latter half of the year, it is noteworthy that the functionalities employed in the composition of case reports with the assistance of ChatGPT were confined to information retrieval, editing, result interpretation, and addressing open-ended inquiries. Notably, there was an absence of utilization for capabilities such as context generation or structural presentation.

### 4.3. Limitation of ChatGPT Mentioned in Case Reports

**Figure 5:**
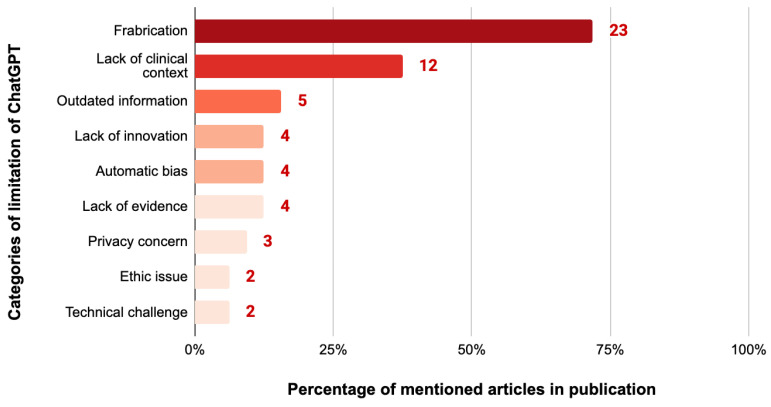
Percentage and number of case report articles categorized by the limitation of ChatGPT in case report writing (N = 32).

Of the 32 articles as reported in Table 1, 23 (71.9%) mentioned inaccuracies in the results generated by ChatGPT, particularly with regard to reference materials which were often fabricated. Twelve articles (37.5%) pointed out the absence of clinical context in ChatGPT, leading to limitations in critical thinking. Five (15.6%) noted that the database only extended up to 2021, rendering it unable to provide the latest data. Four (12.5%) expressed concerns about the lack of creativity in ChatGPT, citing issues like repetitive content, a lack of hypotheses, and a lack of innovation. Similarly, four (12.5%) indicated the potential presence of bias and lack of evidence. Three (9.4%) mentioned privacy concerns. Ethical concerns and the technical challenge were each mentioned by two (6.3%) of the articles, including system crashes that hindered login and inaccuracies in the execution of ChatGPT commands (Figure 5). The limitations mentioned in the latter articles included the inability to interpret medical images, to provide precise numerical values, and to generate accurate responses about rare medical conditions.

## 5. Discussion

The healthcare sector has long been a focal point for the application of artificial intelligence, and the recent introduction of ChatGPT has sparked a flurry of diverse applications [15,16,17,18,19,20,21]. The integration of ChatGPT in medical case report writing has unearthed a number of considerations, extending from the assessment to the broader implications of artificial intelligence in medical documentation. This discussion is dedicated to a thorough analysis of ChatGPT’s role in medical case report generation, highlighting both its utility and limitations.

In our study, we found that ChatGPT is most commonly used for information retrieval in case report writing. The prevalence of this trend remained consistent, underscoring its crucial role as the primary requirement in case report writing. Most of the included articles mentioned using the information retrieval approach by “entering single keywords”, a method similar to conventional search engine or database queries. In an era marked by information explosion and the ever-evolving landscape of medicine, accessing the latest discoveries and accurate, well-organized reference materials in real time has become increasingly challenging for clinicians. The extensive use of information retrieval underscores the medical community’s urgent need for a comprehensive and easily accessible database. However, it is important to recognize that ChatGPT is a large language model, which is inherently different from traditional search engines or medical databases. The content generation process of ChatGPT relies on extensive training on vast textual data, using the deep learning architecture of Transformers to predict the subsequent word based on the provided context. Unlike traditional search engines, which retrieve, aggregate, and selectively present literature based on relevance and quality ratings with real-time updates, ChatGPT does not undergo a literature evaluation process. Furthermore, it also lacks the specialized medical expertise inherent in curated medical databases. Its absence of specialized medical training may result in generalized answers, or in some cases, even lead to fictitious answers, which is also termed “AI hallucination”. Therefore, while ChatGPT serves as a valuable tool for information retrieval, it should be complemented with critical evaluation and verification of the retrieved data to ensure the accuracy and reliability of case reports.

In the domain of ChatGPT-assisted case report writing in our research, content generation was the second most popular application. Notably, the majority of users favored this application for creating draft content and introductions. This trend underscores ChatGPT’s utility in enhancing the efficiency of the writing process, particularly in the initial stages of medical documentation, and highlights the importance of ChatGPT in streamlining the early phases of case report development. It is also worth acknowledging that while ChatGPT’s content generation capabilities offer efficiency gains, they should be viewed as a complementary tool rather than a replacement for professional medical expertise. In addition, this study observed concerns among the included articles regarding the ethical and privacy considerations associated with the use of ChatGPT in medical writing. Similar discussions and recommendations regarding the role of ChatGPT and disclosure practices have also been explored in other research efforts. Given the necessity in case report writing to provide comprehensive narratives of patients’ journeys from symptom onset to medical intervention, these considerations assume an even greater importance. Furthermore, user feedback suggests that clear and specific prompts enhance the relevance of ChatGPT-generated content [34]. Common techniques include providing instructions, contextual information, style and tone, and technical guidance. Continuously refining prompt techniques and iteratively revising ChatGPT-generated content enables authors to achieve desired outcomes more effectively when using ChatGPT for case report writing.

The examination of the temporal trends reveals that the publication of articles was primarily concentrated during the months of April and May, a pattern that does not correspond to the expected trajectory of the adoption of ChatGPT. Considering the observation that the majority of articles were published in the *Cureus* journal, this phenomenon can be attributed to the journal’s proactive solicitation of submissions specifically addressing the utilization of ChatGPT during that period. This discrepancy suggests that despite the potential integration of ChatGPT into academic writing processes, authors may have overlooked the explicit acknowledgment of its involvement in the absence of incentive mechanisms or explicit guidelines mandating the disclosure of AI tool usage. Notably, the articles published in the second half of the year exhibited an absence of the context generation functionality, which had previously ranked second in terms of prevalence. This observation potentially implies that content generation may not possess a sustained role in academic writing endeavors, indicating a potential transitory nature of this capability’s relevance within the scholarly domain.

However, this study also revealed notable limitations in the use of ChatGPT, primarily concerning inaccuracies and the absence of a clinical context. It is worth noting that nearly 70% of the articles reviewed emphasized the issue of inaccuracy when using ChatGPT to write case reports. This concern underscores a critical aspect of medicine, where precision and accuracy are of paramount importance and highlight the potential risks associated with over-reliance on artificial intelligence for medical writing tasks that require high precision and domain-specific expertise. In this situation, prior research recommends that the final output should still be examined by professionals [35,36,37]. The reason for this lies in the inherent limitations of ChatGPT as a language model. Although ChatGPT can comprehend input content, it may not be capable of complex clinical reasoning, such as critical thinking [38]. Given that case reports often involve documenting the course of rare or emerging diseases, the organization of the thinking process and related reasoning becomes even more critical. Moreover, some studies have suggested that precise and appropriate prompts can significantly enhance the accuracy of ChatGPT [39]. The challenge in using ChatGPT, therefore, lies in balancing the efficiency gained with the need for accuracy and contextually relevant information, especially in a field as nuanced and complex as medicine.

The linguistic and cultural implications of using ChatGPT in medical writing are particularly relevant given the journal’s international focus [40,41,42]. Our analysis suggests that while ChatGPT shows promise in aiding medical writing across different linguistic contexts, there are challenges in ensuring the cultural appropriateness and specificity of the content it generates. This is especially pertinent in non-English speaking regions, where nuanced understanding and contextualization of medical information are crucial.

In educational settings, our findings suggest that ChatGPT can serve as a valuable tool for teaching medical writing. Its ability to assist in the organization and structuring of medical narratives offers significant potential for use in the medical field and professional development programs. However, it is essential to approach this integration with caution, ensuring that the use of artificial intelligence complements, rather than replaces, the development of critical writing skills among especially younger healthcare professionals.

The ethical and privacy concerns associated with using ChatGPT in medical writing are non-negligible. Our study indicates a need for stringent guidelines and protocols to safeguard patient data and ensure ethical compliance in the use of artificial intelligence tools like ChatGPT. Given the sensitivity of medical information, it is imperative that these concerns are addressed to maintain trust and integrity in medical documentation.

ChatGPT represents a significant advancement in the realm of medical writing assessments. Its potential for automating certain aspects of the writing process holds promise for improving efficiency. However, our analysis suggests that there is a need for further refinement in its application, particularly in ensuring the accuracy and relevance of the content it generates.

Case studies included in our research provide practical insights into the use of ChatGPT in real-world medical writing scenarios. These examples highlight both the potential benefits and the limitations of artificial intelligence in this context, offering valuable learning points for future applications.

The advancement of artificial intelligence in deep learning has been impressive, especially in the realm of generative tools, extending beyond text processing to include image and language recognition and generation. Following the release of ChatGPT, subsequent developments include Google’s Bard, the new version of GPT-4, Microsoft’s Bing, and Copilot. Bard, developed by Google, can provide multiple draft versions at once and convert generated content into Google online products. Bing functions more like a search engine in a browser, capable of integrating with image generation tools to produce images directly. GPT-4 allows file uploads and collaboration with external websites, such as querying literature or comparing booking information, and can even generate Excel files directly [43]. The ongoing advancement of artificial intelligence holds promise to overcome the limitations mentioned in this study. By connecting to the internet to address database limitations, the development enhances the improvement of content accuracy and traceability. Processing uploaded documents through prompts significantly improves laborious manual transcription and statistical execution. However, these artificial intelligence models still lack transparency in their reasoning logic, resembling black boxes in their computational processes. Furthermore, the companies responsible for these databases have not disclosed their sources, which raises the possibility of bias. This observation underscores the necessity for further development of large language models that are specifically tailored for medical applications to improve their accuracy and relevance for medical purposes. Additionally, they could analyze relevant factors for risk prediction or deliver personalized health education in role-based settings, considering diverse demographic factors such as age, education level, and linguistic and cultural backgrounds [40,41,42].

This study has several limitations. First, it examined only English-language articles from the PubMed database, potentially missing articles in other languages or alternative databases. Second, data collection was confined to articles published up to December 2023, which may have excluded articles still in the process of writing or undergoing review due to publication delays. Moreover, some authors or journals may have chosen not to disclose their use of ChatGPT in medical literature due to ethical concerns, thereby resulting in the omission of potential samples from our study. In addition, certain articles lacked explicit details about their use, or the limitations encountered in practice with ChatGPT, which presented challenges in the analysis process. In addition, the awareness of ChatGPT and its functionalities is constantly evolving. Most of the original articles did not mention when the analysis began and the extent of the author’s understanding of ChatGPT. Furthermore, it is important to note that this study is not based on extensive statistical data, so there may be some variations in the analysis of ChatGPT’s applications and limitations with regard to the broader population. However, it is imperative to acknowledge the absence of a standardized and widely accepted classification framework for the capabilities and limitation of ChatGPT. Consequently, the categorization of applications employed in our study may diverge from the classifications adopted by other researchers, as this domain lacks a universally recognized gold standard.

Finally, our study contributes to the discourse on critical literacy and program evaluation in medical writing. ChatGPT’s role in these areas is complex, as it offers both opportunities for enhancing the accessibility and efficiency of medical documentation, while also presenting challenges in maintaining the depth and accuracy required in medical narratives.

## 6. Conclusions

This comprehensive analysis of ChatGPT’s application in medical case reports reveals a nuanced landscape. While ChatGPT offers significant benefits in terms of efficiency and support in the writing process, especially in medical information retrieval and context generation, its limitations, particularly in terms of accuracy and clinical relevance, cannot be overlooked. Additionally, it is challenging to avoid potential biases when used in the medical field. The ethical and privacy concerns raised by the use of ChatGPT also require further consideration; transparency in disclosing the extent of ChatGPT’s involvement and citing sources is paramount. While ChatGPT can undoubtedly be a valuable tool in medical case reporting, physicians must be aware of its limitations. The findings underscore the importance of combining artificial intelligence tools like ChatGPT with professional expertise and critical thinking. To achieve an evaluation and adaptation of ChatGPT in the medical field will be essential to fully realize the potential of artificial intelligence in enhancing the quality and efficiency of medical documentation.

## Figures and Tables

**Table 1:** Characteristics of case reports using ChatGPT published in PubMed from November 2022 to December 2023.

Characteristics	No. (Percentage)
**Language employed when using ChatGPT**	
English	65 (98.5%)
Spanish	1 (1.5%)
**S** **pecified usage of ChatGPT**	
Mentioned in content	66 (100%)
**Limitation of ChatGPT**	
Mentioned in content	32 (48.5%)
Not mentioned	34 (51.5%)
**Version of ChatGPT**	
Web version only	64 (97.0%)
Web version with extended function	1 (1.5%)
Web and desktop version	1 (1.5%)
**Specialty of case reports**	
Internal Medicine	14 (21.2%)
Otolaryngology	6 (9.1%)
Dermatology	5 (7.6%)
Neurosurgery	4 (6.1%)
Cardiology	4 (6.1%)
Others	33 (50.0%)
**Published Journal**	
*Cureus*	63 (95.5%)
*Arch. Soc. Esp. Oftalmol.*	1 (1.5%)
*Clin. Case Rep.*	1 (1.5%)
*eNeurologicalSci*	1 (1.5%)

## Data Availability

The datasets generated and analyzed during the current study are available as Supplementary Information. These data include raw data and processed data, which support the findings of this study.

## Supplementary Materials

The following supporting information can be downloaded at: https://www.mdpi.com/article/10.3390/ejihpe14040057/s1.

## References

[1] Hassani H., Silva E.S. (2023). The role of ChatGPT in data science: How AI-assisted conversational interfaces are revolutionizing the field. Big Data Cogn. Comput..

[2] Singh H., Singh A. (2023). ChatGPT: Systematic review, applications, and agenda for multidisciplinary research. J. Chin. Econ. Bus. Stud..

[3] Liu H., Azam M., Bin Naeem S., Faiola A. (2023). An overview of the capabilities of ChatGPT for medical writing and its implications for academic integrity. Health Inf. Libr. J..

[4] Haque M.A. (2023). A brief analysis of ‘ChatGPT’—A revolutionary tool designed by OpenAI. EAI Endorsed Trans. AI Robot..

[5] Zohery M. (2023). ChatGPT in academic writing and publishing: A comprehensive guide. Artificial Intelligence in Academia, Research and Science: ChatGPT as a Case Study.

[6] Rahman M., Terano H.J., Rahman N., Salamzadeh A. (2023). ChatGPT and academic research: A review and recommendations based on practical examples. J. Educ. Manag. Dev. Stud..

[7] Xie Y., Seth I., Hunter-Smith D.J., Rozen W.M., Ross R., Lee M. (2023). Aesthetic surgery advice and counseling from artificial intelligence: A rhinoplasty consultation with ChatGPT. Aesthetic Plast. Surg..

[8] Hopkins A.M., Logan J.M., Kichenadasse G., Sorich M.J. (2023). Artificial intelligence chatbots will revolutionize how cancer patients access information: ChatGPT represents a paradigm-shift. JNCI Cancer Spectr..

[9] Srivastava M. (2023). A day in the life of ChatGPT as an academic reviewer: Investigating the potential of large language model for scientific literature review. OSF Prepr..

[10] Ruksakulpiwat S., Kumar A., Ajibade A. (2023). Using ChatGPT in medical research: Current status and future directions. J. Multidiscip. Healthc..

[11] Liu S., Wright A.P., Patterson B.L., Wanderer J.P., Turer R.W., Nelson S.D., McCoy A.B., Sittig D.F., Wright A. (2023). Using AI-generated suggestions from ChatGPT to optimize clinical decision support. J. Am. Med. Inform. Assoc..

[12] Tenhundfeld N.L. Two Birds with One Stone: Writing a Paper Entitled ‘ChatGPT as a Tool for Studying Human-AI Interaction in the Wild’ with ChatGPT. https://www.researchgate.net/publication/368300523_Two_Birds_With_One_Stone_Writing_a_Paper_Entitled_ChatGPT_as_a_Tool_for_Studying_Human-AI_Interaction_in_the_Wild_with_ChatGPT?channel=doi&linkId=63e118c9c97bd76a82765195&showFulltext=true.

[13] Loh E. (2024). ChatGPT and generative AI chatbots: Challenges and opportunities for science, medicine and medical leaders. BMJ Lead..

[14] Preiksaitis C., Sinsky C.A., Rose C. (2023). ChatGPT is not the solution to physicians’ documentation burden. Nat. Med..

[15] Sirrianni J., Sezgin E., Claman D., Linwood S.L. (2022). Medical text prediction and suggestion using generative pretrained transformer models with dental medical notes. Methods Inf. Med..

[16] Waisberg E., Ong J., Masalkhi M., Kamran S.A., Zaman N., Sarker P., Lee A.G., Tavakkoli A. (2023). GPT-4 and ophthalmology operative notes. Ann. Biomed. Eng..

[17] Singh S., Djalilian A., Ali M.J. (2023). ChatGPT and ophthalmology: Exploring its potential with discharge summaries and operative notes. Semin. Ophthalmol..

[18] Bosbach W.A., Senge J.F., Nemeth B., Omar S.H., Mitrakovic M., Beisbart C., Horváth A., Heverhagen J., Daneshvar K. (2024). Ability of ChatGPT to generate competent radiology reports for distal radius fracture using RSNA template items and integrated AO classifier. Curr. Probl. Diagn. Radiol..

[19] Elkassem A.A., Smith A.D. (2023). Potential use cases for ChatGPT in radiology reporting. AJR Am. J. Roentgenol..

[20] Rau A., Rau S., Zoeller D., Fink A., Tran H., Wilpert C., Nattenmueller J., Neubauer J., Bamberg F., Reisert M. (2023). A context-based chatbot surpasses trained radiologists and generic ChatGPT in following the ACR Appropriateness Guidelines. Radiology.

[21] Patel S.B., Lam K. (2023). ChatGPT: The future of discharge summaries?. Lancet Digit. Health.

[22] Ariyaratne S., Iyengar K.P., Nischal N., Chitti Babu N., Botchu R. (2023). A comparison of ChatGPT-generated articles with human-written articles. Skelet. Radiol..

[23] Au Yeung J., Kraljevic Z., Luintel A., Balston A., Idowu E., Dobson R.J., Teo J.T. (2023). AI chatbots not yet ready for clinical use. Front. Digit. Health.

[24] Barrot J.S. (2023). Using ChatGPT for second language writing: Pitfalls and potentials. Assess. Writ..

[25] Fournier-Tombs E., McHardy J. (2023). A medical ethics framework for conversational artificial intelligence. J. Med. Internet Res..

[26] Dave T., Athaluri S.A., Singh S. (2023). ChatGPT in medicine: An overview of its applications, advantages, limitations, future prospects, and ethical considerations. Front. Artif. Intell..

[27] Eke D.O. (2023). ChatGPT and the rise of generative AI: Threat to academic integrity?. J. Responsib. Technol..

[28] Mijwil M.M., Hiran K.K., Doshi R., Dadhich M., Al-Mistarehi A.-H., Bala I. (2023). ChatGPT and the future of academic integrity in the artificial intelligence era: A new frontier. Al-Salam J. Eng. Technol..

[29] Stokel-Walker C. (2023). ChatGPT listed as author on research papers: Many scientists disapprove. Nature.

[30] Su Y., Lin Y., Lai C. (2023). Collaborating with ChatGPT in argumentative writing classrooms. Assess. Writ..

[31] Zheng H., Zhan H. (2023). ChatGPT in scientific writing: A cautionary tale. Am. J. Med..

[32] Yang J., Chen Y.-L., Por L.Y., Ku C.S. (2023). A systematic literature review of information security in chatbots. Appl. Sci..

[33] Nakagawa K., Moukheiber L., Celi L.A., Patel M., Mahmood F., Gondim D., Hogarth M., Levenson R. (2023). AI in pathology: What could possibly go wrong?. Semin. Diagn. Pathol..

[34] Meskó B. (2023). Prompt Engineering as an Important Emerging Skill for Medical Professionals: Tutorial. J. Med. Internet Res..

[35] Vaishya R., Misra A., Vaish A. (2023). ChatGPT: Is this version good for healthcare and research?. Diabetes Metab. Syndr. Clin. Res. Rev..

[36] Hosseini M., Rasmussen L.M., Resnik D.B. (2023). Using AI to write scholarly publications. Account. Res..

[37] Seth I., Rodwell A., Tso R., Valles J., Bulloch G., Seth N. (2023). A conversation with an open artificial intelligence platform on osteoarthritis of the hip and treatment. J. Orthop. Sports Med..

[38] Ricci F.L., Consorti F., Pecoraro F., Luzi D., Tamburis O. (2022). A Petri-Net-Based Approach for Enhancing Clinical Reasoning in Medical Education. IEEE Trans. Learn. Technol..

[39] Lingard L. (2023). Writing with ChatGPT: An illustration of its capacity, limitations & implications for academic writers. Perspect. Med. Educ..

[40] Sallam M., Salim N., Barakat M., Al-Tammemi A. (2023). ChatGPT applications in medical, dental, pharmacy, and public health education: A descriptive study highlighting the advantages and limitations. Narra J..

[41] Van Bulck L., Moons P. (2024). What if your patient switches from Dr. Google to Dr. ChatGPT? A vignette-based survey of the trustworthiness, value and danger of ChatGPT-generated responses to health questions. Eur. J. Cardiovasc. Nurs..

[42] Gupta R., Herzog I., Weisberger J., Chao J., Chaiyasate K., Lee E.S. (2023). Utilization of ChatGPT for plastic surgery research: Friend or foe?. J. Plast. Reconstr. Aesthet. Surg..

[43] Oh N., Choi G.S., Lee W.Y. (2023). ChatGPT goes to the operating room: Evaluating GPT-4 performance and its potential in surgical education and training in the era of large language models. Ann. Surg. Treat. Res..

